# An outcome-based definition of low birthweight for births in low- and middle-income countries: a secondary analysis of the WHO global survey on maternal and perinatal health

**DOI:** 10.1186/s12887-019-1546-z

**Published:** 2019-05-27

**Authors:** Malinee Laopaiboon, Pisake Lumbiganon, Siwanon Rattanakanokchai, Warut Chaiwong, João Paulo Souza, Joshua P. Vogel, Rintaro Mori, Ahmet Metin Gülmezoglu

**Affiliations:** 1 Department of Epidemiology and Biostatistics, Faculty of Public Health, Khon Kaen University, 123 Mittraphap Road, Nai-Muang, Muang District, Khon Kaen, 40002 Thailand; 2 Department of Obstetrics and Gynaecology, Faculty of Medicine, Khon Kaen University, Khon Kaen, Thailand; 3 Bangkok Health Research Center 2 Soi Soonvijai 7, New Petchburi Rd., Huaykwang, Bangkok, 10310 Thailand; 4 Department of Social Medicine, Ribeirão Preto Medical School, University of São Paulo, Ribeirão Preto, SP Brazil; 5 UNDP • UNFPA • UNICEF • WHO • World Bank Special Programme of Research, Development and Research Training in Human Reproduction, Department of Reproductive Health and Research, World Health Organization, Geneva, Switzerland; 6 Maternal and Child Health Program, Burnet Institute, 85 Commercial Road, Melbourne, 3004 Australia; 7 Department of Health Policy, National Center for Child Health and Development, Tokyo, Japan; 8 Department of Reproductive Health and Research World Health Organization, Avenue Appia 20, CH-1211 Geneva 27, Switzerland

**Keywords:** Low birthweight, Outcome-based definition, Early neonatal mortality

## Abstract

**Background:**

2500 g has been used worldwide as the definition of low birthweight (LBW) for almost a century. While previous studies have used statistical approaches to define LBW cutoffs, a LBW definition using an outcome-based approach has not been evaluated. We aimed to identify an outcome-based definition of LBW for live births in low- and middle-income countries (LMICs), using data from a WHO cross-sectional survey on maternal and perinatal health outcomes in 23 countries.

**Methods:**

We performed a secondary analysis of all singleton live births in the WHO Global Survey (WHOGS) on Maternal and Perinatal Health, conducted in African and Latin American countries (2004–2005) and Asian countries (2007–2008). We used a two-level logistic regression model to assess the risk of early neonatal mortality (ENM) associated with subgroups of birthweight (< 1500 g, 1500–2499 g with 100 g intervals; 2500–3499 g as the reference group). The model adjusted for potential confounders, including maternal complications, gestational age at birth, mode of birth, fetal presentation and facility capacity index (FCI) score. We presented adjusted odds ratios (aORs) with 95% confidence intervals (CIs). A lower CI limit of at least two was used to define a clinically important definition of LBW.

**Results:**

We included 205,648 singleton live births at 344 facilities in 23 LMICs. An aOR of at least 2.0 for the ENM outcome was observed at birthweights below 2200 g (aOR 3.8 (95% CI; 2.7, 5.5) of 2100–2199 g) for the total population. For Africa, Asia and Latin America, the 95% CI lower limit aORs of at least 2.0 were observed when birthweight was lower than 2200 g (aOR 3.6 (95% CI; 2.0, 6.5) of 2100–2199 g), 2100 g (aOR 7.4 (95% CI; 5.1, 10.7) of 2000–2099 g) and 2200 g (aOR 6.1 (95% CI; 3.4, 10.9) of 2100–2199 g) respectively.

**Conclusion:**

A birthweight of less than 2200 g may be an outcome-based threshold for LBW in LMICs. Regional-specific thresholds of low birthweight (< 2200 g in Africa, < 2100 g in Asia and < 2200 g in Latin America) may also be warranted.

**Electronic supplementary material:**

The online version of this article (10.1186/s12887-019-1546-z) contains supplementary material, which is available to authorized users.

## Background

The term low birthweight (LBW) is defined by the World Health Organization (WHO) as the weight at birth of a neonate less than 2500 g (g), a cut-off that is often consistent with 10th percentile for gestation [1, 2]. This cut-off was based on epidemiological observations that neonates of birthweight less than 2500 g were more likely to die than heavier newborns [3], with mortality rates rising rapidly as birthweight decreases [4–6]. WHO advises that the 2500 g cut-off value should be used for international health statistics comparisons [1].

Low birthweight is an important public health indicator of maternal malnutrition and health, and poor antenatal care [1, 3]. Globally, more than 20 million newborns (an estimated 15.5% of all births) are born low birthweight each year. More than 95% of these LBW neonates are born in low- and middle-income countries (LMICs) [1]. There is significant variation of LBW rates among geographical regions. The highest LBW rates are seen in Asia (18.3%), about three times higher than the lowest rate, in Europe (6.4%). There is considerable variation between sub-regions in Asia, ranging from 5.9% in Eastern Asia to 27% in South-central Asia [1, 7].

LBW has been associated with increased risks of neonatal mortality and several neonatal morbidities, including birth asphyxia, acute respiratory infections and diarrhea disease, as well as longer-term adverse health outcomes such as neurological disorders, impaired language development, poor academic performance, cardiovascular disease and diabetes [3, 8, 9]. Decreasing the global burden of LBW could substantially reduce costs to families and o healthcare systems in LMICs [10].

However, some evidence has emerged that the cut-off value of 2500 g to define LBW may not be appropriate for all settings. For example, some countries such as Sri Lanka have a high prevalence of neonates with birthweight less than 2500 g do not have correspondingly high neonatal mortality rates [11]. To this effect, WHO suggested that individual countries should adopt a population-specific cut-off value for LBW to guide clinical care [1]. However, in practice a birthweight below 2500 g is still in routine use in most LMICs.

Defining an appropriate LBW cut-off is challenging. If too low, some neonates may not get necessary care. Alternatively, if the value is too high, some neonates may get additional care that is not necessary. In many LMICs, inappropriate use of limited health resources can disadvantage neonates requiring more intensive care. Therefore, further investigation of the most appropriate cut-off for LBW remains an important issue.

Previous studies have defined population-specific cut-offs for LBW in high-income countries (HICs) [12, 13] and LMICs [14, 15]. This is typically done using statistical methods, where the lowest 10th percentile of the birthweight distribution is used as the cut-off for LBW. These has often resulted in LBW cut-offs higher than 2500 g – for example, 2750 g in the US in 1992 [12] to 3000 g in Denmark in 2007 [13]. Recent studies in LMICs have also identified alternative LBW cut-offs, such as 2600 g in a study in sub-urban Cameroon [14] and 2700 g in a rural community [15] in Cameroon.

Previous studies have used an outcome-based approach for identification of cut-off weights for fetal growth [16] and macrosomia [17]. By using the Health Statistics database for the years 1995–2002 of the United State National Center, Joseph et al. generated fetal growth standards for singleton and twin neonates based on severe morbidity and mortality outcomes [16]. In a secondary analysis of the database of the World Health Organization (WHO) Global Survey on Maternal and Perinatal Health (2004–2008) conducted in 23 LMICs in Africa, Asia, and Latin America, Ye et al. defined macrosomia based on the adjusted assoiated risk of birthweight for maternal and perinatal mortality and morbidity in term pregnancies [17]. However, we have identified no previous analyses that have defined a LBW cut-off value using an outcome-based approach. This study therefore aimed to identify an outcome-based definition of LBW for LMICs using the WHO Global Survey database.

## Methods

### Study design and population

We conducted a secondary analysis using data from the WHO Global Survey (WHOGS) on Maternal and Perinatal Health conducted in Africa, Asia and Latin America. The WHOGS was a prospective, facility-based, cross-sectional survey on maternal and perinatal health interventions and outcomes. The primary aim of the survey was to assess the association between mode of birth and maternal and perinatal health outcomes [18, 19]. Details of the survey have been reported elsewhere [18–20]. A total of 373 facilities in 24 countries in three regions participated in this survey. Data collection was performed in 2004–05 for Africa and Latin America, and in 2007–08 for Asia. Trained data collectors reviewed medical records of individual women from the time of attending participating facilities for delivery until discharge, death or day 7 postpartum. Data were abstracted from medical records into structured case record forms. The period of data collection was two months in facilities with at least 6000 deliveries per year and three months in facilities with less than 6000 deliveries per year. Institutional data were collected for each participating facility, including information on available resources for obstetric care. The data was obtained through an interview with the hospital director or head of obstetrics, and data entered into the pre-specified institutional form.

The WHOGS protocol was approved by the WHO ethics review committee and the relevant local review committees for all participating centres [19]. Individual informed consent was not obtained; survey data were extracted from medical records without individual identification or patient contact [19]. We received permission to use this data from the Department of Reproductive Health and Research, WHO on January 14th, 2014.

**Fig. 1:**
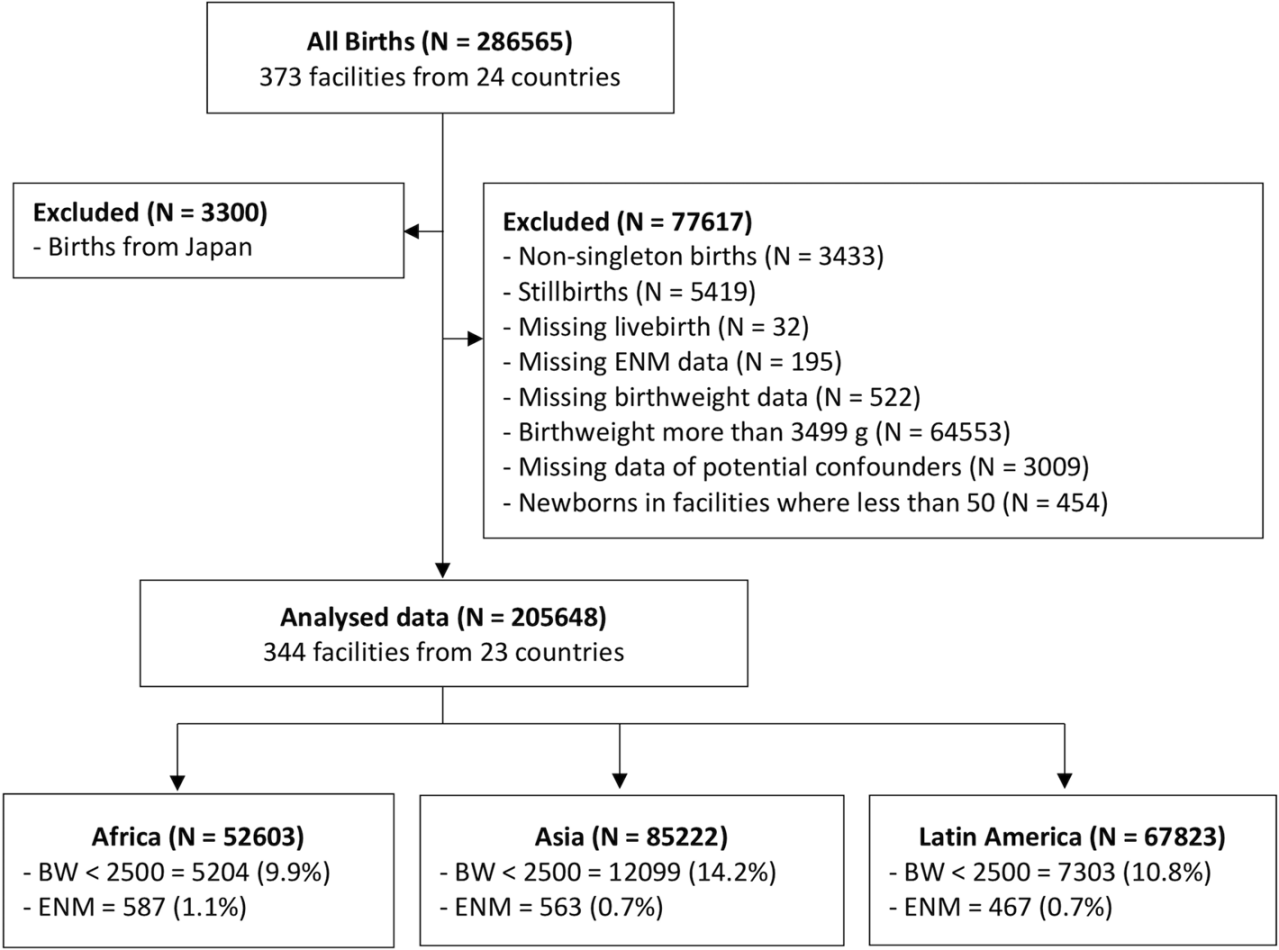
Flow chart of inclusion and exclusion of study neonates

Our analysis was restricted to singleton, live newborns in participating facilities in low- and middle-income countries (facilities and newborns in Japan were excluded). We aimed to evaluate associations between birthweight cut- offs and early neonatal mortality (ENM). ENM was defined as death occurring in hospital prior to discharge or Day 7 (whichever came first). We adjusted for potential confounders, including maternal complications (such as chronic hypertension, sickle cell anaemia) gestational age at birth, mode of birth and fetal presentation at birth. Newborns with missing information on birthweight, ENM outcome and potential confounders were excluded. We also excluded facilities that had less than 50 newborns [21]. The selection process for the analysis population is shown in Fig. 1.

We used data on the availability of basic and essential maternal healthcare services of individual participating facilities as potential confounding factors at facility level. Facilities were classified into different levels, using the existing WHOGS facility capacity index (FCI) score [18]. FCI scores ranged between 0 and 16. Facilities with a total score of 9 or less were defined as low capacity, those with scores of between 10 and 12 as medium capacity, and those with scores of 13 or more as high capacity [18].

### Statistical analysis

We assessed the association of birthweight groups with ENM using two-level logistic regression models. We assigned facilities to represent units at level two and individuals within facilities at level one. We used the birthweight range of 2500–3499 g as the reference group based on the current global clinical practice for normal neonatal birthweight range. In addition, the rates of ENM at 100 g intervals within 2500 – 3499 g were quite similar (around 0.5% in our database) [see Additional file 1]. We classified birthweights of 1500–2499 g into 100 g intervals. Birthweights less than 1500 g were classified into one group. We adjusted for potential confounders at both levels in the models (see above). We estimated adjusted odds ratios (ORs) and 95% confidence intervals (CIs) of ENM by absolute birthweight subgroups. We analysed all associations in the whole database and by region (Africa, Asia and Latin America).

It is well-known that the risk of adverse neonatal outcomes increases as birthweight decreases; very low birthweight infants (1500 g or less) are at greatest risk [22–25]. We applied this concept in our analysis to identify an appropriate low birthweight cut-off based on the ENM. We used an a priori odds ratio threshold of 2.0 (for the lower confidence interval) as a criterion for clinical significance, as per previous studies [17, 26, 27]. Thus, in this analysis the clinical significance was defined as when the lower limit of the 95% confidence interval for the adjusted OR was at least 2.0 [28]. We, therefore, defined LBW from the lowest birthweight of the subgroup that its lower subgroups had lower limit of the 95% confidence interval for adjusted OR was at least 2.0 [28].

The descriptive analyses were also done using R software. We used package lme4 of R software to analyse the two-level logistic regression model [29].

## Results

A total of 205,648 singleton live newborns at 344 facilities in 23 LMICs were included in this analysis (Fig. 1). There were differences in the birthweight distribution for the three regions. Mean birthweights were 2935 g (SD 389 g) in Africa, 2838 g (SD 406 g) in Asia and 2958 g (SD 428 g) in Latin America. The rates of birthweight < 2500 g were 9.9, 14.2 and 10.8%, in Africa, Asia and Latin America respectively. The rates of birthweight < 1500 g were 0.8, 0.8 and 1.4%, in Africa, Asia and Latin America respectively. Wide variation of birthweight was observed between Asian countries (Table 1).

**Table 1:** Country-specific birthweight and early neonatal mortality distribution of singleton liveborn births

Countries	Number of facilities	Number of newborns	Birthweight (g)				Early Neonatal Mortality (%)
			Mean	(SD)	< 1500 (%)	< 2500 (%)	
Total	344	205,648	2902	(413)	1.0	12.0	0.8
Africa	118	52,603	2935	(389)	0.8	9.9	1.1
Algeria	18	7516	3025	(390)	1.0	7.4	1.3
Angola	15	3920	2903	(405)	1.2	11.8	0.5
Congo	21	6767	2874	(392)	0.6	14.4	1.0
Kenya	20	13,586	2921	(395)	1.0	9.7	1.8
Niger	11	6526	2906	(381)	0.5	11.6	0.4
Nigeria	21	5704	2932	(380)	0.9	8.2	0.8
Uganda	12	8584	2964	(363)	0.6	7.7	1.1
Asia	112	85,222	2838	(406)	0.8	14.2	0.7
Cambodia	5	4457	2897	(383)	0.8	9.3	1.1
China	21	9357	3045	(332)	0.4	5.5	0.3
India	20	22,008	2653	(405)	1.2	22.1	1.1
Nepal	8	6852	2786	(386)	0.5	13.7	0.9
Philippines	17	11,732	2819	(419)	1.3	16.2	1.1
Sri Lanka	14	13,191	2846	(381)	0.6	15.5	0.2
Thailand	12	8068	2939	(380)	0.7	10.8	0.4
Viet Nam	15	9557	2997	(335)	0.3	5.7	0.1
Latin America	114	67,823	2958	(428)	1.4	10.8	0.7
Argentina	14	6661	2975	(453)	1.9	11.0	0.5
Brazil	19	10,955	2956	(437)	1.5	11.6	0.8
Cuba	17	8300	3013	(379)	0.7	8.0	0.2
Ecuador	14	9666	2898	(432)	1.5	12.4	0.7
Mexico	21	15,716	2932	(429)	1.5	11.6	0.6
Nicaragua	6	4271	2946	(384)	0.7	9.5	1.0
Paraguay	6	2001	2990	(442)	1.6	10.4	1.1
Peru	17	10,253	2997	(438)	1.7	9.8	1.0

We present the associations between birthweight intervals and potential confounding factors in Table 2. Mean gestational age was positively associated with birthweight. Higher rates of all potential confounding factors were seen among infants with lower birthweights. In the study population, the caesarean section rate was 24.3 and 16.9% of women had a maternal complication.

**Table 2:** Distribution of individual potential confounding factors by birthweight

Birthweight (g)	n	Gestational age		Cesarean section	Breech presentation at birth	Maternal complications^a^
		Mean	(SD)	(%)	(%)	(%)
Overall	205,648	38.5	(2.0)	49,964 (24.3)	9099 (4.4)	34,642 (16.9)
< 1500	2072	30.6	(4.1)	38.9	18.6	25.3
1500–1599	602	33.1	(3.2)	32.4	11.1	20.3
1600–1699	521	33.7	(3.1)	39.3	12.1	22.5
1700–1799	811	34.4	(3.0)	35.8	12.1	20.3
1800–1899	973	34.6	(2.9)	35.9	10.4	22.1
1900–1999	1032	35.2	(2.9)	36.7	9.5	23.3
2000–2099	2807	36.7	(2.6)	26.7	7.5	15.7
2100–2199	2165	36.9	(2.5)	29.7	7.3	19.2
2200–2299	3817	37.2	(2.3)	26.2	6.4	15.6
2300–2399	4178	37.6	(2.0)	25.5	5.2	18.2
2400–2499	5628	37.9	(2.0)	25.9	5.5	18.7
2500–3499	181,042	38.8	(1.6)	23.7	3.9	16.6

All variables differed significantly by birthweight categories (*p*-value < 0.001)

^a^Maternal complications included chronic hypertension, cardiac disease, renal disease, chronic respiratory condition, diabetes mellitus, malaria, sickle cell anaemia, severe anaemia, pyelonephritis or urinary infection, HIV/AIDS, Thalassemia and other medical conditions

ENM rates were 1.1, 0.7 and 0.7% in Africa, Asia and Latin America respectively. When compared to the reference (normal birthweight) group (2500–3499 g), adjusted ORs of ENM show statistical significance when birthweight was lower than 2500 g in the total population and each region. However, adjusted ORs of ENM in birthweights of 2300–2399 g in Africa (aOR 1.7 (95% CI; 0.9, 3.3)) and Asia (aOR 1.3 (95% CI; 0.6, 2.7)) did not reach statistical significance.

**Fig. 2:**
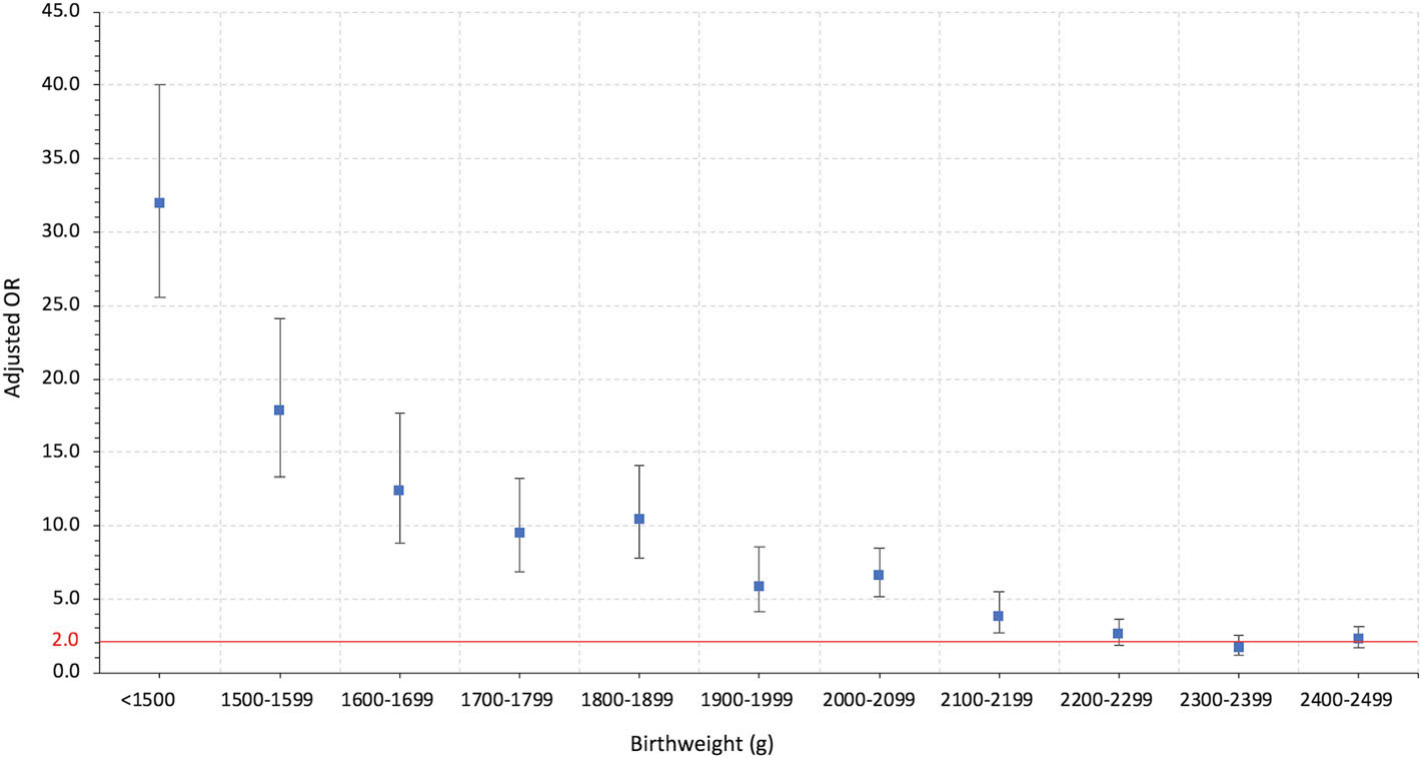
Adjusted odds ratios with 95% CI of early neonatal mortality by birthweight in the total population. Solid red line shows the adjusted OR 2.0 for clinical significance

The adjusted ORs of ENM were similar (about 2.0) for birthweight intervals 2400-2499 g and 2200-2299 g, compared to the reference group. The adjusted ORs gradually increased from 3.8 (95% CI; 2.7, 5.5) for birthweights of 2100–2199 g to 17.9 (95% CI; 13.3, 24.1) in birthweights of 1500–1599 g (Fig. 2). The adjusted OR increased up to 32.0 (95% CI; 25.6, 40.0) in birthweight < 1500 g (Table 3). Based on the pre-defined clinical significance criterion for aOR (lower limit of 95% CI of 2.0), the LBW cut-off was 2200 g for the total population.

**Table 3:** Rate and adjusted odds ratios of early neonatal mortality by birthweight

Birthweight (g)	n	Early Neonatal Mortality^a^	
		Rate (%)	Adjusted OR (95% CI)
< 1500	2072	29.0	32.0 (25.6, 40.0)
1500–1599	602	13.8	17.9 (13.3, 24.1)
1600–1699	521	9.6	12.5 (8.8, 17.7)
1700–1799	811	6.2	9.5 (6.8, 13.2)
1800–1899	973	6.8	10.5 (7.8, 14.1)
1900–1999	1032	3.6	5.9 (4.1, 8.5)
2000–2099	2807	3.1	6.6 (5.2, 8.5)
2100–2199	2165	1.6	3.8 (2.7, 5.5)
2200–2299	3817	1.0	2.6 (1.9, 3.7)
2300–2399	4178	0.6	1.7 (1.1, 2.6)
2400–2499	5628	0.7	2.3 (1.7, 3.1)
2500–3499	181,042	0.3	1.0

Adjusted for gestational age, mode of delivery, fetal presentation at delivery, maternal complications and complexity index

^a^*ROC* = 0.9118

When compared to the reference group (2500–3499 g), the adjusted ORs of ENM in birthweight of < 2500 g were high across all three regions, similar to the total population. However, the adjusted ORs of ENM in birthweight of < 1500 g in Asia reached 54.3 (95% CI; 37.4, 78.9) while those in Africa and Latin America was 30.4 (95% CI; 21.1, 43.7) and 18.1 (95% CI; 11.5, 28.5), respectively (Table 4).

**Table 4:** Rate and adjusted odds ratios of early neonatal mortality by birthweight and regions

Birthweight (g)	Early neonatal mortality								
	Africa^a^ (*n* = 52,603)			Asia^b^ (*n* = 85,222)			Latin America^c^ (*n* = 67,823)		
	n	Rate (%)	Adjusted OR (95% CI)	n	Rate (%)	Adjusted OR (95% CI)	n	Rate (%)	Adjusted OR (95% CI)
< 1500	435	36.8	30.4 (21.1, 43.7)	677	28.5	54.3 (37.4, 78.9)	960	25.8	18.1 (11.5, 28.5)
1500–1599	114	22.8	20.6 (11.9, 35.7)	327	11.9	23.1 (14.9, 36.0)	161	11.2	13.9 (7.5, 25.5)
1600–1699	116	14.7	11.0 (6.0, 20.4)	209	7.7	15.6 (8.5, 28.5)	196	8.7	12.4 (6.8, 22.8)
1700–1799	143	7.7	6.4 (3.2, 12.7)	387	5.9	13.4 (8.2, 22.0)	281	5.7	8.8 (4.8, 16.1)
1800–1899	175	12.6	11.5 (6.8, 19.5)	458	7.0	16.3 (10.6, 25.2)	340	3.5	5.2 (2.7, 10.3)
1900–1999	228	6.6	6.5 (3.7, 11.6)	431	2.8	7.4 (3.9, 13.9)	373	2.7	4.4 (2.2, 9.0)
2000–2099	636	4.1	5.5 (3.6, 8.6)	1571	2.7	7.4 (5.1, 10.7)	600	3.2	8.3 (4.8, 14.3)
2100–2199	481	2.7	3.6 (2.0, 6.5)	999	0.8	2.7 (1.3, 5.6)	685	2.0	6.1 (3.4, 10.9)
2200–2299	682	2.2	3.0 (1.8, 5.2)	2225	0.7	2.3 (1.4, 4.0)	910	0.9	3.0 (1.4, 6.3)
2300–2399	901	1.1	1.7 (0.9, 3.3)	2043	0.3	1.3 (0.6, 2.7)	1234	0.6	2.6 (1.3, 5.5)
2400–2499	1293	1.3	2.2 (1.3, 3.6)	2772	0.5	2.2 (1.3, 3.7)	1563	0.6	3.0 (1.5, 5.8)
2500–3499	47,399	0.5	1.0	73,123	0.2	1.0	60,520	0.1	1.0

Adjusted for gestational age, mode of delivery, fetal presentation at delivery, maternal complications and complexity index

^a^*ROC* = 0.8804

^b^*ROC* = 0.9101

^c^*ROC* = 0.9245

**Fig. 3:**
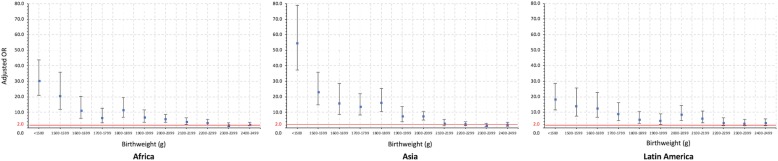
Adjusted odds ratios with 95% CI of early neonatal mortality by birthweight in the three regions. Solid red line shows the adjusted OR 2.0 for clinical significance

With regards to the pre-defined clinical significance criterion, in Africa the adjusted OR was 3.6 (95% CI; 2.0, 6.5) in birthweights of 2100–2199 g. In Asia, the adjusted OR was 7.4 (95% CI; 5.1, 10.7) in birthweights of 2000–2099 g. In Latin America, the adjusted OR was 6.1 (95% CI; 3.4, 10.9) in birthweights of 2100-2199 g. Therefore, the LBW cut-offs were 2200 g, 2100 g and 2200 g for Africa, Asia and Latin America respectively (Fig. 3).

## Discussion

Our findings show that a low birthweight cut-off based on ENM outcome is 2200 g for a large, multi-country population of live singleton newborns. The low birthweight cut-off at regional level were similar, 2200 g, 2100 g and 2200 g for Africa, Asia and Latin America respectively. These LBW cut-offs are lower than the traditional criterion of 2500 g. The risks of ENM were quite similar among newborns in with birthweight ranges of 2200–2299 g, 2300–2399 g and 2400–2499 g with adjusted ORs around two, but their lower limit of the 95% confidence intervals did not reach our pre-specified criterion for clinical significance.

Although the a priori adjusted OR of 2.0 for ENM was arbitrarily set as clinically important, the value has been used in previous studies. Ye et al. [17] used the OR of 2.0 to define macrosomia that is clinically significant risk for maternal and perinatal mortality and morbidity in LMICs, also using the WHOGS database. Boulet et al. [27] also used this value for defining clinically important fetal growth restriction. Barrette et al. [26] used the inverse value of 2.0 relative risk (0.5) to clinically justify difference between the planned caesarean delivery and vaginal delivery in the randomized trial of the Twin Birth Study Collaborative Group. However, previous studies [17, 26, 27] did not report whether the clinically significant OR of 2.0 was identified with respect to the lower 95% confidence interval. This concept has been suggested by Mccluskey [28] in which clinical significance of any data has to be above or below the range of confidence interval that shows statistical significance. We consider these findings to be reliable in identify the clinically important outcome-based definition of low birthweight using ENM as a primary outcome.

The findings of this analysis are outcome-based criteria for which the definition of low birthweight was identified from the association model between birthweight and ENM adjusted for important confounding factors, maternal complications, gestational age at birth, mode of birth, fetal presentation and facility complexity index. The analyses were performed in the large, multi-country dataset of the WHO Global Survey [18]. Our findings based on the outcome-based approach done in the large database may be more appropriate than those based on statistical criteria [14, 30–34]. For example, Brimblecombe [30] suggested the classification of low birthweight based on birthweight distributional components. This study proposed two Gaussian distributions to describe birthweight - the primary distribution was composed of the majority of birthweights, whereas the secondary distribution was the minority of high-risk birthweights centered at the lower tail of the primary distribution. In 1980 Rooth proposed a definition of low birthweight based on a cut-off of weights less than two standard deviations below the local population mean, that better predicted risk for neonatal mortality [31]. Wilcox and Russell proposed an approach to explain association between birthweight and perinatal mortality. They suggested three parameters should define birthweight characteristics of a population: 1) mean and 2) standard deviation of the Gaussian distribution that included between 95 and 98% of term birthweight population, and 3) the proportion of all births in the residual distribution that mostly consisted of small preterm birth [32–34]. Recently, Njim et al. [14] conducted a two-phased observational study to set a clinical cut-off point for LBW and to assess its incidence, predictors and complications in a sub-urban hospital in Cameroon. The authors found 2600 g was the cut-off at the 10th centile of birth weight for low birthweight. The cut-off point provided significant higher incidence of low birthweight (19%) than that of the traditional cut-off of 2500 g (13.5%). They also showed that newborns with birthweights between 2500 g and 2600 g had significant higher rates of complications than those with birthweights > 2600 g in the study population. Agbor et al. [15] also performed a study with a similar objective and method to Njim et al.’s paper in a rural sub-division in Cameroon. They assessed the statistical LBW cut-off at the 10th percentile of the observed birthweights distribution. They also made the comparison of neonatal adverse outcomes between LBW (birthweight <10th percentile) and heavier neonates (birthweight ≥10th percentile) in the study population for assessing the clinical significance. The authors reported the clinical cut-off point for LBW at 2700 g in the rural community in Cameroon. They found 6.1% of neonates had birthweights between 2500 g and 2700 g, with higher stillbirth rate (about 3 %, 5/163) than those of heavier neonates (< 1 %, 12/1553).

In our findings the odds of ENM clearly increased for every 100 g reduction of birth weight after 2200 g. Malin et al. reported a similar finding in a systematic review - a birthweight less than 1500 g had the highest odds of neonatal mortality (OR 48.6, 95% CI 28.62, 82.53). Increasing the birthweight cut-off point to 2000 g, 2500 g or 2900 g gradually reduced the risk, but the summary estimates remained highly significant at each cut-off point [14]. This review did not report the risk of neonatal mortality by a narrower birthweight range (100 g each) as we did.

This study was a secondary analysis of the WHO Global Survey database conducted in 23 countries across Africa, Asia and Latin America. Trained personnel systematically collected the data. In the analyses, we controlled for important confounding factors of ENM such as maternal complications, gestational age at delivery, and mode of birth. However, the WHOGS database was primarily aimed at evaluating different modes of delivery and pregnancy outcomes, rather than to explore newborn birthweights specifically. The WHOGS was a facility-based survey, performed in large, secondary and tertiary facilities where caesarean section was available. Our findings might lead to over-representation of neonatal adverse outcomes and consequently might not reflect the situation in smaller facilities. There might be errors in birthweight data due to variations between facilities in the quality of birthweight measurement. For example, medical personnel might preferentially report birthweight values ending in a rounded number (0 or 5) which may affect the study findings. Our primary outcome focused only on early neonatal death occurring prior to discharge or day 7; information was not available on late neonatal or infant deaths. The cross-sectional study design only permits us to evaluate associations rather than causation.

## Conclusions

Our analysis suggests that the outcome-based definition of LBW of less than 2200 g may be used instead of the conventional less than 2500 g for assessing BW risk for early neonatal mortality. A regional specific definition of low birthweight (< 2200 g in Africa, < 2100 g in Asia and < 2200 g in Latin America) are quite similar and may be more appropriate for each region.

## Additional file

Additional file 1: The percentage of early neonatal mortality by 100 g interval of birthweights. The rates of ENM among 100 g intervals of these birthweights are quite similar of around 0.5% in our analysed database. (PDF 185 kb)

## Data Availability

The datasets generated and/or analyzed during the current study are not publicly available due to they belonged to Department of Reproductive Health and Research, The World Health Organization but could be available from WHO on reasonable request.

## Abbreviations

aORs: Adjusted odds ratios
CIs: Confidence intervals
ENM: Early neonatal mortality
FCI: Facility capacity index
g: Grams
kg: Kilograms
LBW: Low birthweight
LMICs: Low and middle income countries
ORs: Odds ratios
WHO: World Health Organization
WHOGS: WHO Global Survey

## References

[1] Wardlaw T, World Health Organization, UNICEF (2004). Low birthweight: country regional and global estimates..

[2] World Health Organization (1992). ICD-10: international statistical classification of diseases and related health problems.

[3] Kramer MS (1987). Determinants of low birth weight: methodological assessment and meta-analysis. Bull World Health Organ.

[4] Chase HC (1969). Infant mortality and weight at birth: 1960 United States birth cohort. Am J Public Health Nations Health.

[5] Ghosh S (1982). Weight of all births and infant mortality. J Epidemiol Community Health.

[6] Saugstad LF (1981). Weight of all births and infant mortality. J Epidemiol Community Health.

[7] Cutland CL, Lackritz EM, Mallett-Moore T, Bardaji A, Chandrasekaran R, Lahariya C (2017). Low birth weight: case definition & guidelines for data collection, analysis, and presentation of maternal immunization safety data. Vaccine..

[8] Zerbeto AB, Cortelo FM, Élio Filho BC (2015). Association between gestational age and birth weight on the language development of Brazilian children: a systematic review. J Pediatr.

[9] Badshah S, Mason L, McKelvie K, Payne R, Lisboa PJ (2008). Risk factors for low birthweight in the public-hospitals at Peshawar, NWFP-Pakistan. BMC Public Health.

[10] Sicuri E, Bardají A, Sigauque B, Maixenchs M, Nhacolo A, Nhalungo D (2011). Costs associated with low birth weight in a rural area of southern Mozambique. PLoS One.

[11] Pathmanathan I, Liljestrand J, Martins JM, Rajapaksa LC, Lissner C, de Silva A, et al. Investing in maternal health: learning from Malaysia and Sri Lanka. Washington, DC: The World Bank; 2003.

[12] Goldenberg RL, Hoffman HJ, Cliver SP, Cutter GR, Nelson KG, Copper RL (1992). The influence of previous low birth weight on birth weight, gestational age, and anthropometric measurements in the current pregnancy. Obstet Gynecol.

[13] Rode L, Hegaard HK, Kjærgaard H, Møller LF, Tabor A, Ottesen B (2007). Association between maternal weight gain and birth weight. Obstet Gynecol.

[14] Njim T, Atashili J, Mbu R, Choukem SP (2015). Low birth weight in a sub-urban area of Cameroon: an analysis of the clinical cut-off, incidence, predictors and complications. BMC Pregnancy Childbirth.

[15] Agbor VN, Ditah C, Tochie JN, Njim T. Low birthweight in rural Cameroon: Q5 515 an analysis of a cut-off value. BMC Pregnancy Childbirth. 2018;18. 10.1186/s12884-018-1663-y. 10.1186/s12884-018-1663-y PMC5769287 29334919

[16] Joseph KS, Fahey J, Platt RW, Liston RM, Lee SK, Sauve R (2009). An outcome-based approach for the creation of fetal growth standards: do singletons and twins need separate standards?. Am J Epidemiol.

[17] Ye J, Torloni MR, Ota E, Jayaratne K, Pileggi-Castro C, Ortiz-Panozo E (2015). Searching for the definition of macrosomia through an outcome-based approach in low- and middle-income countries: a secondary analysis of the WHO global survey in Africa, Asia and Latin America. BMC Pregnancy Childbirth..

[18] Lumbiganon P, Laopaiboon M, Gülmezoglu AM, Souza JP, Taneepanichskul S, Ruyan P (2010). Method of delivery and pregnancy outcomes in Asia: the WHO global survey on maternal and perinatal health 2007-08. Lancet..

[19] Shah A, Faundes A, Machoki M, Bataglia V, Amokrane F, Donner A (2008). Methodological considerations in implementing the WHO global survey for monitoring maternal and perinatal health. Bull World Health Organ.

[20] Souza JP, Cecatti JG, Faundes A, Morais SS, Villar J, Carroli G (2010). Maternal near miss and maternal death in the World Health Organization’s 2005 global survey on maternal and perinatal health. Bull World Health Organ.

[21] Maas CJM, Hox JJ (2005). Sufficient sample sizes for multilevel modeling. Methodology..

[22] Wilcox A, Skjaerven R, Buekens P, Kiely J (1995). Birth weight and perinatal mortality. A comparison of the United States and Norway. JAMA..

[23] Graafmans WC, Richardus JH, Borsboom GJJM, Bakketeig L, Langhoff-Roos J, Bergsjø P (2002). Birth weight and perinatal mortality: a comparison of “optimal” birth weight in seven Western European countries. Epidemiology..

[24] Vangen S, Stoltenberg C, Skjaerven R, Magnus P, Harris JR, Stray-Pedersen B (2002). The heavier the better? Birthweight and perinatal mortality in different ethnic groups. Int J Epidemiol.

[25] Habib NA, Dalveit AK, Mlay J, Oneko O, Shao J, Bergsjø P (2008). Birthweight and perinatal mortality among singletons and twins in North-Eastern Tanzania. Scand J Public Health.

[26] Barrett JFR, Willan AR, Joseph KS (2014). Planned cesarean or vaginal delivery for twin pregnancy. N Engl J Med.

[27] Boulet SL, Alexander GR, Salihu HM, Kirby RS, Carlo WA (2006). Fetal growth risk curves: defining levels of fetal growth restriction by neonatal death risk. Am J Obstet Gynecol.

[28] Mccluskey A, Lalkhen AG (2007). Statistics IV: interpreting the results of statistical tests. Contin Educ Anaesthesia, Crit Care Pain.

[29] Bates D, Maechler M, Bolker B, Walker SW (2015). Fitting linear mixed-effects models using lme4. J Stat Softw.

[30] Brimblecombe FS, Ashford JR (1968). Significance of low birth weight in perinatal mortality. A study of variations within England and Wales. Br J Prev Soc Med.

[31] Rooth G (1980). Low birthweight revised. Lancet (London, England).

[32] Wilcox AJ, Russell IT (1983). Perinatal mortality: standardizing for birthweight is biased. Am J Epidemiol.

[33] Wilcox AJ, Russell IT (1986). Birthweight and perinatal mortality: III. Towards a new method of analysis. Int J Epidemiol.

[34] Wilcox AJ, Russell IT (1983). Birthweight and perinatal mortality: I. on the frequency distribution of birthweight. Int J Epidemiol.

