# Supplementary figures and images for: An outcome-based definition of low birthweight for births in low- and middle-income countries: a secondary analysis of the WHO global survey on maternal and perinatal health
Source: BMC Pediatr. 2019 May 27;19:166. doi: 10.1186/s12887-019-1546-z (PMC6535858; doi:10.1186/s12887-019-1546-z)

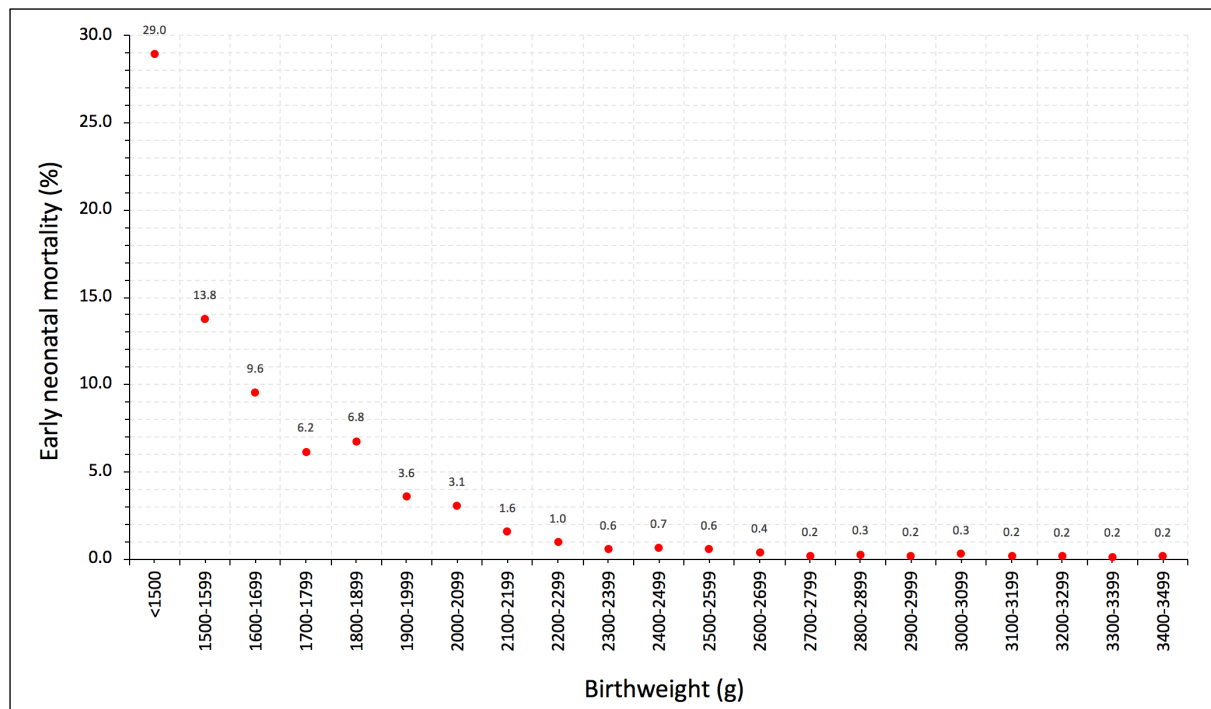

**Additional file 1** The percentage of early neonatal mortality by 100 g interval of birthweights

Supplement: Supplementary file 1 — The percentage of early neonatal mortality by 100 g interval of birthweights. The rates of ENM among 100 g intervals of these birthweights are quite similar of around 0.5% in our analysed database. (PDF 185 kb) [file 12887_2019_1546_MOESM1_ESM.pdf]
